# CD4^+^NKG2D^+^ T Cells Exhibit Enhanced Migratory and Encephalitogenic Properties in Neuroinflammation

**DOI:** 10.1371/journal.pone.0081455

**Published:** 2013-11-25

**Authors:** Tobias Ruck, Stefan Bittner, Catharina C. Gross, Johanna Breuer, Stefanie Albrecht, Sabrina Korr, Kerstin Göbel, Susann Pankratz, Christian M. Henschel, Nicholas Schwab, Ori Staszewski, Marco Prinz, Tanja Kuhlmann, Sven G. Meuth, Heinz Wiendl

**Affiliations:** 1 Department of Neurology, University of Münster, Münster, Germany; 2 Interdisciplinary Center for Clinical Research (IZKF), Münster, Münster, Germany; 3 Institute of Neuropathology, University of Münster, Münster, Germany; 4 Institute of Neuropathology and BIOSS Centre for Biological Signaling Studies, University of Freiburg, Freiburg, Germany; 5 Institute of Physiology I - Neuropathophysiology, University of Münster, Münster, Germany; Hannover Medical School, Germany

## Abstract

Migration of encephalitogenic CD4^+^ T lymphocytes across the blood-brain barrier is an essential step in the pathogenesis of multiple sclerosis (MS). We here demonstrate that expression of the co-stimulatory receptor NKG2D defines a subpopulation of CD4^+^ T cells with elevated levels of markers for migration, activation, and cytolytic capacity especially when derived from MS patients. Furthermore, CD4^+^NKG2D^+^ cells produce high levels of proinflammatory IFN-γ and IL-17 upon stimulation. NKG2D promotes the capacity of CD4^+^NKG2D^+^ cells to migrate across endothelial cells in an in vitro model of the blood-brain barrier. CD4^+^NKG2D^+^ T cells are enriched in the cerebrospinal fluid of MS patients, and a significant number of CD4^+^ T cells in MS lesions coexpress NKG2D. We further elucidated the role of CD4^+^NKG2D^+^ T cells in the mouse system. NKG2D blockade restricted central nervous system migration of T lymphocytes in vivo, leading to a significant decrease in the clinical and pathologic severity of experimental autoimmune encephalomyelitis, an animal model of MS. Blockade of NKG2D reduced killing of cultivated mouse oligodendrocytes by activated CD4^+^ T cells. Taken together, we identify CD4^+^NKG2D^+^ cells as a subpopulation of T helper cells with enhanced migratory, encephalitogenic and cytotoxic properties involved in inflammatory CNS lesion development.

## Introduction

Multiple sclerosis (MS) and its animal model, experimental autoimmune encephalomyelitis (EAE), are chronic inflammatory disorders of the central nervous system (CNS) characterized by inflammation, demyelination, and axonal degeneration. The pathogenesis of MS is thought to be an autoimmune process particularly mediated by the adaptive immune system [1]. It is commonly assumed that myelin-specific autoreactive effector T cells that have been primed in secondary lymphoid tissues migrate into the CNS where they are re-activated and initiate the inflammatory cascade [2]. T cell activation requires both antigen-specific TCR (T cell receptor) as well as co-stimulatory signaling. The co-stimulatory signal is provided by accessory molecules, including B7 family members [3] or NKG2D (natural-killer group 2, member D, CD314) [4] that both play important roles in various pathologic processes [5,6].

NKG2D is an activating (co)stimulating receptor expressed on various lymphoid and myeloid cell types with a preferential expression on NK cells, CD8^+^ T cells and γδ T cells in humans and mice [7,8]. Furthermore, a small subpopulation of CD4^+^ T cells (referred to as CD4^+^NKG2D^+^ T cells) with frequencies of 0.5–3% can be detected in the peripheral blood of healthy individuals [9]. CD4^+^NKG2D^+^ T cells have been reported to play a pathogenic role in autoimmune disorders such as Crohn’s disease [6] or rheumatoid arthritis (RA) [10] and their respective animal models [11,12]. These CD4^+^NKG2D^+^ cells are expanded in the peripheral blood and especially in the respective target organs. Furthermore, they exert TCR-independent cytotoxic activity against NKG2D ligand-expressing target cells and have been shown to produce Th1 and Th17 cytokines both in RA and in Crohn’s disease [6,10,12,13]. In contrast, a previous study [14] also suggested an immunoregulatory role of CD4^+^NKG2D^+^ T cells with predominant production of IL-10 under polyclonal stimulation. 

NKG2D interacts with various ligands: human MICA/B (MHC class I chain-related protein A and B) and ULBP1–6 (UL16-binding proteins 1–6) or mouse Rae1 (retinoic acid early transcript 1), H60 (histocompatibility 60) and Mult1 (mouse UL16-binding protein-like transcript 1) [8,15,16]. The ligands are upregulated by cellular stress including viral infection, inflammation, and transformation, indicating a physiologic role in immune responses to various cellular hazards [17,18]. Interestingly, Saikali et al. [19] showed that oligodendrocytes express the NKG2D ligands MICA/B in white matter sections obtained from multiple sclerosis lesions, but not in healthy control samples. Furthermore, these authors proposed that an NKG2D-NKG2D ligand interaction between cytotoxic immune cells and oligodendrocytes potentially contributes to selective cell death in these cells. However, further knowledge about the role of the NKG2D in the pathophysiology of MS is still lacking.

We here demonstrate that NKG2D expression on CD4^+^ T lymphocytes (CD3^+^CD4^+^CD8^-^CD56^-^) is strongly associated with markers of cell migration and effector functions and that this inflammatory CD4^+^ subset produces high levels of inflammatory Th1 or Th17 cytokines. In a human BBB (blood-brain barrier) model, NKG2D promotes the propensity of CD4^+^NKG2D^+^ T cells to migrate across endothelial cells, indicating an important role for NKG2D in T cell CNS migration. Accordingly, numbers of CD4^+^NKG2D^+^ T cells are elevated in the cerebrospinal fluid (CSF) and in CNS lesions of MS patients. CD4^+^NKG2D^+^ T cells in the CSF of MS patients show a shift towards an effector memory phenotype compared to healthy controls. Blockade of NKG2D in vivo leads to a significant decrease in the clinical and pathologic severity in myelin oligodendrocyte protein (MOG)-peptide induced EAE. Furthermore, blockade of NKG2D reduces the cytotoxicity of activated T cells towards cultivated mouse oligodendrocytes in vitro.

## Results

### CD4^+^NKG2D^+^ T cells are characterized by enhanced levels of markers for migration, activation, and cytolytic capacity, and produce pro-inflammatory cytokines

**Figure 1:**
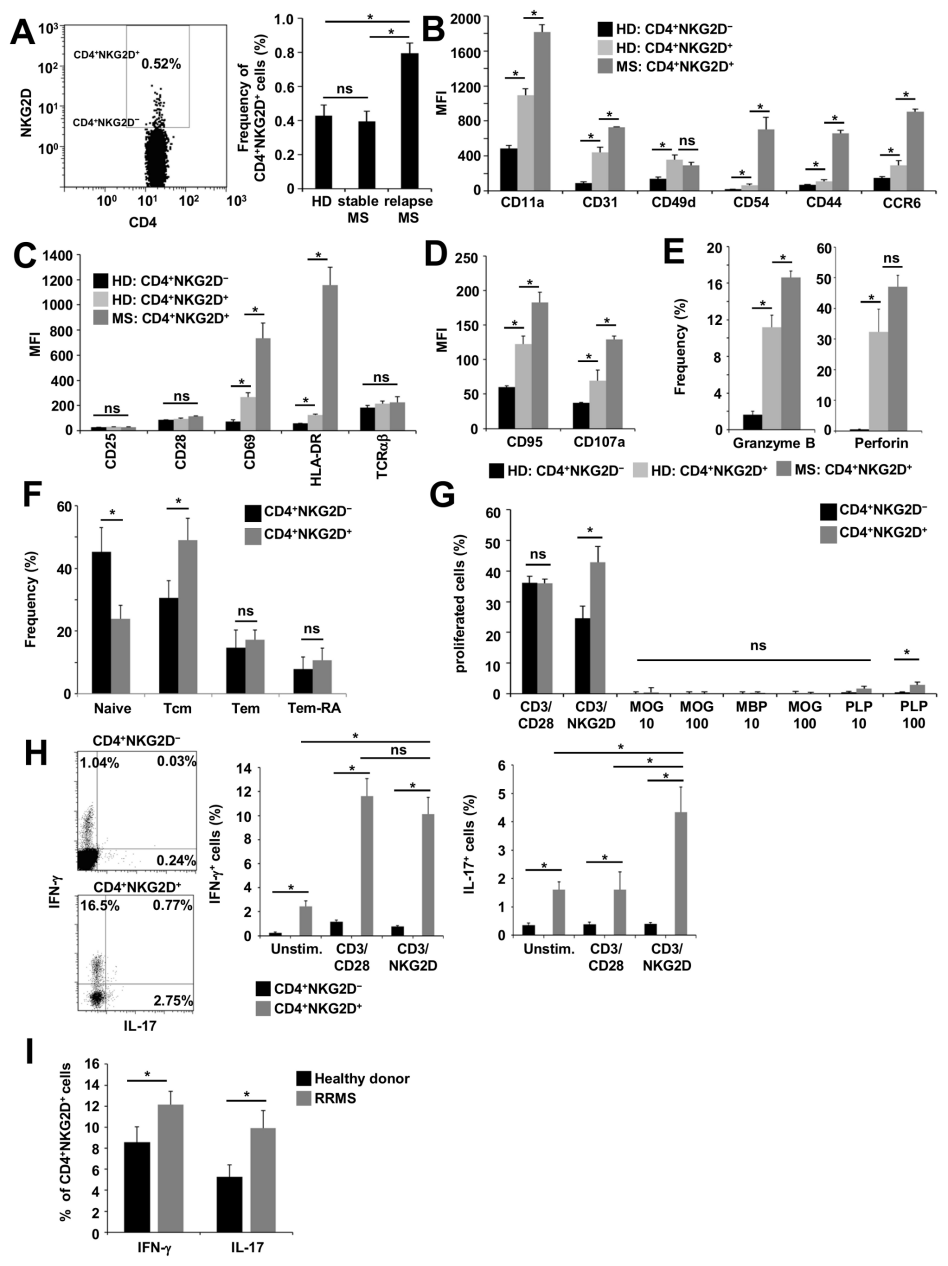
CD4^+^NKG2D^+^ T cells exert pro-migratory, cytolytic and pro-inflammatory properties. (**A**) Flow cytometry: The dot plot shows a representative example of NKG2D expression on CD3^+^CD4^+^CD8^-^CD56^−^ T cells derived from the peripheral blood of a healthy donor (HD). The bar graph represents the mean frequency of CD4^+^NKG2D^+^ T cells in the peripheral blood of HDs (*n* = 20), stable RRMS (*n* = 15) and active RRMS patients (*n* = 14). (**B**–**E**) Mean fluorescence intensity (MFI) of different markers indicative for migratory capacity (**B**), activation (**C**), or cytolytic capacity (**D**, **E**) of CD4^+^NKG2D^+^ and CD4^+^NKG2D^−^ T cells from the peripheral blood of HDs (n = 6) or active RRMS patients (*n* = 6). (**F**) Percentages of naive (CD45RA^+^CD62L^+^), T central memory (Tcm, CD45RA^-^CD62L^+^), T effector memory (Tem, CD45RA^-^CD62L^−^) and T effector memory RA (Tem-RA, CD45RA^+^CD62L^-^) cells in the CD4^+^NKG2D^+^ and CD4^+^NKG2D^−^ T cell compartment assessed by flow cytometry (*n* = 6 HDs). (**G**) Carboxyfluorescein succinimidyl ester (CFSE) proliferation assays of CD4^+^NKG2D^+^ T cells and CD4^+^NKG2D^−^ T cells under CD3/CD28, CD3/NKG2D, MOG_35-55_ (10 µg/ml or 100 µg/ml), MBP_1-11_ or PLP_190-209_ stimulation (*n* = 8 HDs). (**H**) Intracellular cytokine staining for IFN-γ and IL-17 of CD4^+^NKG2D^+^ and CD4^+^NKG2D^−^ T cells derived from the peripheral blood of HDs (*n* = 7). The dot plots depict a representative example of IFN-γ- and IL-17-positive cells upon CD3/CD28 stimulation. The bar graphs show the frequencies of IFN-γ or IL-17 positive cells of unstimulated, CD3/CD28- or CD3/NKG2D-stimulated cells. (**I**) Comparison of the proportions of IFN-γ or IL-17 positive CD3/CD28-stimulated CD4NKG2D^+^ T cells derived from frozen PBMCs of HDs (*n* = 6) or active RRMS patients (*n* = 6). *P < 0.05. ns, not significant; unstim., unstimulated.

The expression of the co-stimulatory molecule NKG2D on CD4^+^ T cells is restricted to a small subset in the peripheral blood of healthy donors. Patients with stable RRMS (relapsing-remitting multiple sclerosis) showed comparable levels, whereas active RRMS patients demonstrated elevated frequencies of CD4^+^NKG2D^+^ T cells (Figure 1A) in the periphery. A pathogenic role has been postulated for CD4^+^NKG2D^+^ T cells under autoinflammatory conditions, raising the question of whether this distinct immune cell subset might be involved in the pathophysiology of MS. In accordance with its putative pro-inflammatory functions, freshly isolated CD3^+^CD4^+^CD8^-^CD56^-^NKG2D^+^ T cells (further referred to as CD4^+^NKG2D^+^ within this article, for the gating strategy see Figures S1A and S1B) from healthy controls showed significantly higher levels of markers of migration (Figure 1B), activation (Figure 1C), and cytolytic capacity (Figure 1D, E) in healthy donors. Interestingly CD4+NKG2D+ T cells derived from the peripheral blood of treatment-naïve, RRMS patients with high disease activity expressed even higher levels of these markers. NKG2D^-^ CD4^+^ T cells from healthy donors and RRMS patients showed no significant differences in the expression of the above mentioned markers except for CCR6 (Figure S3C-F). Furthermore, CD4^+^NKG2D^+^ T cells from healthy donors were characterized by a shift to a central memory phenotype (Figure 1F). Under CD3/NKG2D stimulation CD4^+^NKG2D^+^ T cells showed significantly higher proliferation rates in comparison with CD4^+^NKG2D^−^ T cells. No differences in cell proliferation were observed for CD3/28, MOG_35-55_ (myelin oligodendrocyte glycoprotein), MBP_1-11_ (myelin basic protein) and low dose PLP_190-209_ (proteolipid protein) stimulation (Figure 1G and Figure S3G). Only high doses of PLP_190-209_ (100 µg/ml) led to significantly increased proliferative responses. Most importantly, CD4^+^NKG2D^+^ T cells produced significantly higher levels of IFN-γ and IL-17 than CD4^+^NKG2D^−^ T cells both under basal conditions and upon CD3/CD28 stimulation (Figure 1H). Of note, co-stimulation of NKG2D yielded comparable amounts of IFN-γ production and even higher levels of IL-17 production compared with classical CD28 co-stimulation alone (Figure 1H). Using peripheral blood of treatment-naive, stable RRMS patients, CD4^+^NKG2D^+^ T cells produced significantly higher levels of IFN-γ and IL-17 upon stimulation when compared with cells from healthy controls (Figure 1I). 

### CD4^+^NKG2D^+^ T cells exhibit enhanced migratory properties at the blood-brain barrier and show enrichment in the CSF and in CNS lesions of MS patients

**Figure 2:**
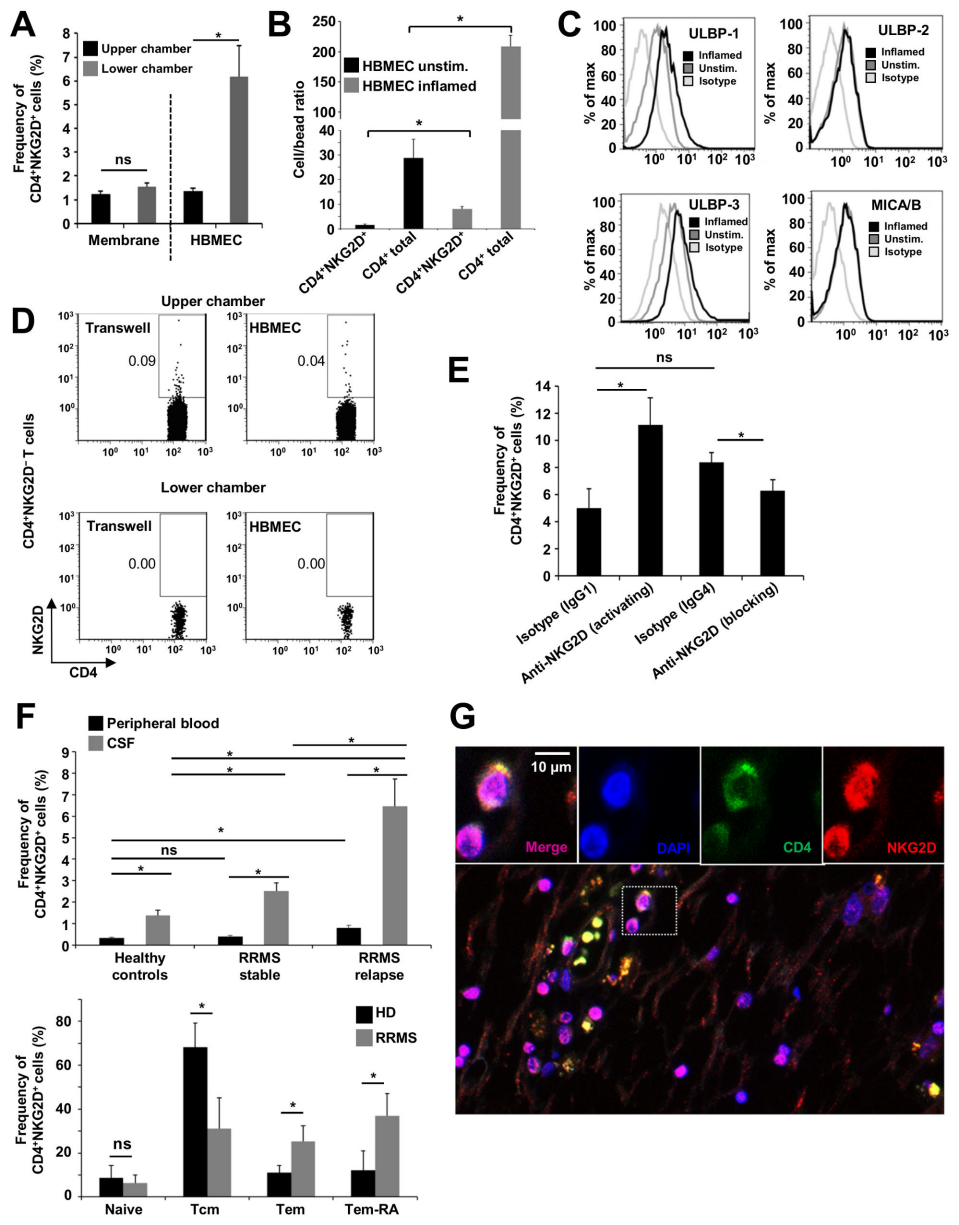
NKG2D facilitates migration of CD4^+^NKG2D^+^ T cells in vitro and increased numbers are found in CSF and lesions of MS patients. (**A**) Transmigration Assay of CD4^+^ T cells from healthy donors (HDs; *n* = 5) for a migration period of 11–12 h. Migration through the membrane only (membrane) or over an endothelial cell single-layer (HBMEC) was compared. The proportions of migrated (lower chamber) or non-migrated (upper chamber) CD4^+^NKG2D^+^ T cells are shown. (**B**) Relative cell numbers of transmigrated CD4^+^NKG2D^+^ T cells and total CD4^+^ T cells through a non-inflamed or inflamed HBMEC single-layer (*n* = 5 HDs). (**C**) Expression of NKG2D ligands on non-inflamed or inflamed HBMECs (500 IU/ml IFN-γ and 500 IU/ml TNF-α for 72 h) analyzed by flow cytometry (representative example; *n* = 5). (**D**) Representative transmigration assay of FACS-sorted CD4^+^NKG2D^−^ T cells. Proportions of migrated (lower chamber) or non-migrated (upper chamber) cells are shown (*n* = 5). (**E**) CD4^+^ T cells from HDs (*n* = 6) were incubated with antibodies (5 μg/ml activating anti-NKG2D or 10 μg/ml blocking anti-NKG2D) or the respective isotype controls prior to the transmigration assay. Proportions of migrated CD4^+^NKG2D^+^ T cells after 6 h are shown. (**F**) Upper Panel: Frequencies of CD4^+^NKG2D^+^ T cells in the peripheral blood and the cerebrospinal fluid (CSF) of patients with stable (*n* = 15) and active (*n* = 14) relapsing-remitting MS (RRMS) and healthy controls (*n* = 15) assessed by flow cytometry. Lower Panel: Percentages of naive (CD45RA^+^CD62L^+^), T central memory (Tcm, CD45RA^-^CD62L^+^), T effector memory (Tem, CD45RA^-^CD62L^−^) and T effector memory RA (Tem-RA, CD45RA^+^CD62L^-^) CD4^+^NKG2D^+^ cells in the CSF of RRMS patients (RRMS, n = 6) and healthy controls (HD, *n* = 6). (**G**) Histopathologic characterization of a representative human MS lesion (patient with RRMS) using antibodies directed against CD4 and NKG2D, a perivascular region is magnified showing CD4^+^NKG2D^+^ T cells (DAPI, blue; CD4, green; NKG2D, red). See also Table 1 for quantification. *P < 0.05. ns, not significant; unstim., unstimulated.

The migration of autoreactive T cells into the CNS across the BBB is a critical step in the pathophysiology of MS. Unstimulated CD4^+^NKG2D^+^ T cells already displayed significantly higher levels of cell adhesion molecules (e.g. CD44, CD49d, LFA-1) and chemokine receptors (e.g. CCR6; Fig. 1B). Next we performed functional assays to assess their migratory capacity in a model of the human BBB in vitro. Using a monolayer of human brain microvascular endothelial cells (HBMECs) mimicking migratory processes at the BBB, CD4^+^NKG2D^+^ T cells were enriched in the lower chamber (Fig. 2A and Fig. S2A). This enhanced migratory capacity could not be observed when migration was performed without HBMECs, pointing towards a specific role for T cell-endothelial cell interactions (Fig. 2A). Inflammatory conditions of the BBB, as seen in MS, were mimicked by pre-stimulation of HBMECs using IFN-γ and TNF-α. Proinflammatory prestimulation led to a significant increase of transmigration of both total CD4^+^ T cells as well as CD4^+^NKG2D^+^ T cells (Fig. 2B). Of note, HBMECs showed no significant lysis by CD4^+^NKG2D^+^ T cells within the time period of migration (Fig. S2B). HBMECs expressed the activating NKG2D ligands ULBP-1, ULBP-2, ULBP-3, and MICA/B (Fig. 2C), supporting the assumption of a specific role of the NKG2D-NKG2D ligand interaction for T cell transmigration. Among these, ULBP-1 and ULBP-3 were upregulated under inflammatory conditions (Fig. 2C). FACS-sorted CD4^+^NKG2D^−^ cells were used in another set of experiments to exclude the possibility of an upregulation of NKG2D on T cells during the migration process. Using this set-up, we could exclude that NKG2D is upregulated during the in vitro adhesion and migration process, since no NKG2D was detectable in the lower compartment (Figure 2D). Furthermore, blocking NKG2D antibodies resulted in a significantly reduced migration of CD4^+^NKG2D^+^ T cells. Interestingly, activating antibodies further increased the migratory potential of CD4^+^NKG2D^+^ cells (Figure 2E), These experiments further support our assumption of a ligand-specific interaction and demonstrate the importance of the NKG2D-signaling pathway for CD4^+^NKG2D^+^ T cell trafficking at the BBB. Corresponding with our in vitro observations, significantly higher frequencies of CD4^+^NKG2D^+^ T cells were found in the CSF compared with the peripheral blood of healthy controls (Figure 2F, upper panel and Figure S3A). Of note, significant differences could only be observed for the peripheral blood of RRMS patients with a relapse. Frequencies of CD8^+^NKG2D^+^ T cells were comparable in the peripheral blood and the CSF of healthy donors and MS patients (Figure S4A). Furthermore CD4^+^NKG2D^+^ cells in the CSF of MS patients showed a shift to an effector memory phenotype compared to healthy controls (Figure 2F, lower panel). This could not be observed in the peripheral blood (Figure S3B).

CD4^+^NKG2D^+^ T cells were also found in perivascular regions of human MS lesions (Figure 2G and Table 1) in higher numbers compared with healthy controls and normal-appearing gray matter of MS patients. As a control for the specific staining of NKG2D nearly all CD8+ T cells expressed NKG2D (Figure S4B). This in vivo data in MS patients and controls support the assumption that CD4^+^NKG2D^+^ T cells exhibit preferential trafficking over the BBB and enhance at sites of CNS inflammation.

**Table 1:** Elevated levels of NKG2D expression in lesions of human MS patients.

**Control (NAWM)**	**CD4^+^ NKG2D^*+*^**	**MS patients (NAWM)**	**CD4^+^ NKG2D^*+*^**	**MS patients (lesion)**	**CD4^+^ NKG2D^*+*^**
Subject 1	**0**	Patient 1	**+**	Patient 1	**++**
Subject 2	**+**	Patient 2	**0**	Patient 2	**+++**
Subject 3	**0**	Patient 3	**0**	Patient 3	**+**
Subject 4	**0**	Patient 4	**0**	Patient 4	**++**
Subject 5	**0**	Patient 5	**+**	Patient 5	**++**

Quantification of CD4^+^NKG2D**^*+*^** expression was performed by two blinded investigators in normal-appearing white matter (NAWM) of healthy donors (left column) or human multiple sclerosis (MS) patients (middle column) and lesions of MS patients (right column). (0, no CD4^+^NKG2D**^*+*^** cells detectable; +, occasional NKG2D**^*+*^**CD4^+^ cells detectable, ++, NKG2D**^*+*^**CD4^+^ cells < 20%; +++, NKG2D**^*+*^**CD4^+^ cells > 20%).

### Inhibition of the NKG2D-signaling pathway ameliorates the disease course of EAE

**Figure 3:**
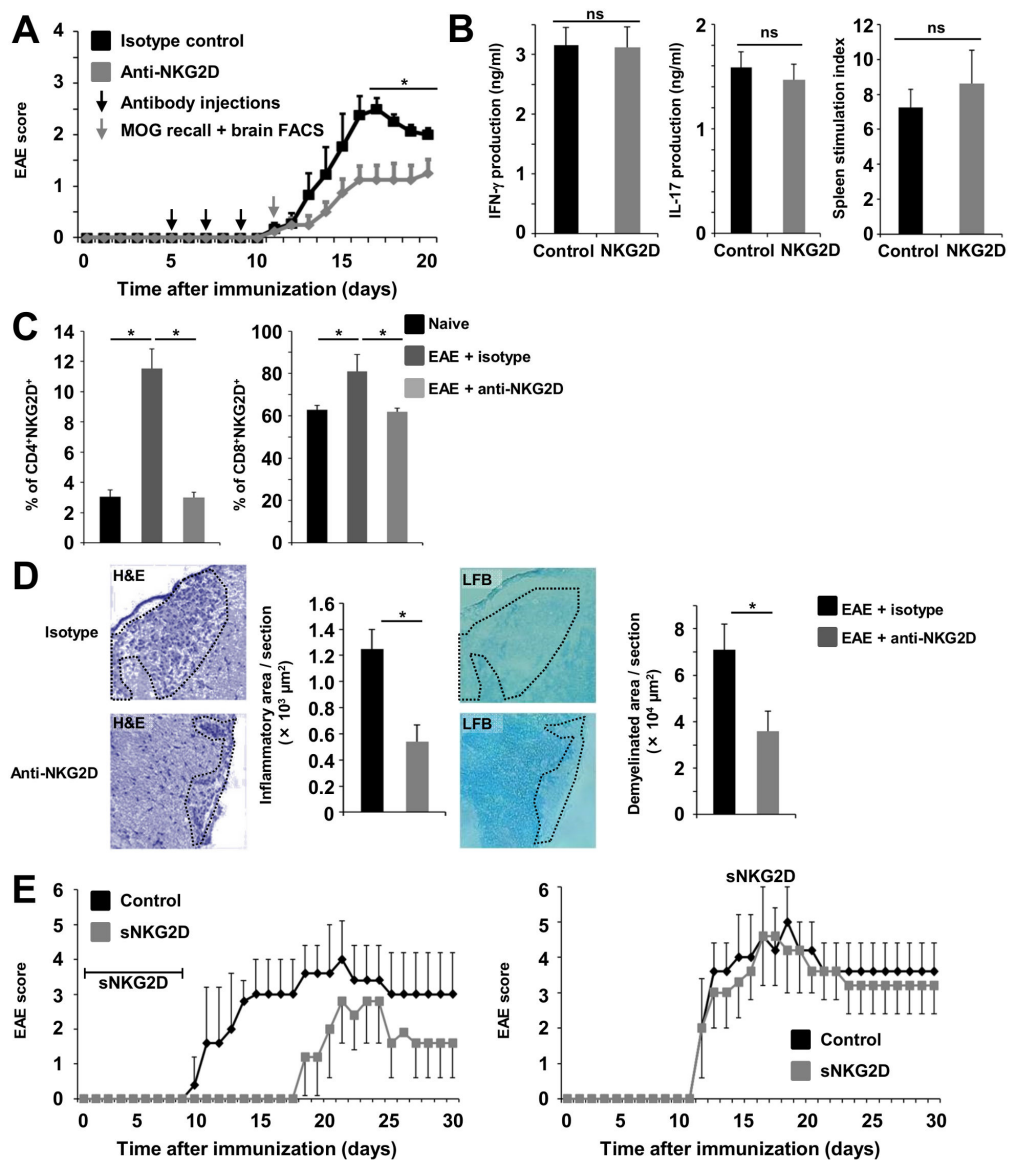
Inhibition of the NKG2D-signaling pathway ameliorates the disease course of experimental autoimmune encephalomyelitis (EAE). (**A**) EAE disease course was reduced in NKG2D-blocking-antibody-treated C57BL/6 mice (black arrows, days of injection, clone CX5) compared with mice treated with an isotype control (two independent experiments, *n* = 8 each). Myelin oligodendrocyte protein (MOG) recall assays and flow cytometry were performed at disease onset (gray arrow). (**B**) ELISAs for IFN-γ production and the splenocyte stimulation index indicative of cell proliferation showed no significant differences for splenocytes of antibody- and isotype-treated mice at disease onset (*n* = 5 mice per group). (**C**) Flow cytometric quantification of NKG2D^+^ CD4^+^ and CD8^+^ lymphocytes invading the brain at disease onset of EAE (*n* = 5 mice per group, clone 7 used for staining). (**D**) Representative immunohistochemical staining of spinal cord samples from isotype- or anti-NKG2D-treated mice (*n* = 10 per mouse; *n* = 5 mice per group) at the disease maximum of EAE and quantification of the area of infiltrating cells (hematoxylin and eosin [H&E] staining, highlighted area) and area of demyelination (luxol fast blue [LFB] staining, highlighted area). (**E**) In vivo blockade of NKG2D by soluble NKG2D (sNKG2D) delayed the onset of EAE when administered before disease onset (left panel). In contrast, the administration of sNKG2D after disease onset had no significant effects (right panel). (*n* = 6 mice per group). *P < 0.05. ns, not significant.

We next assessed the in vivo functional impact of NKG2D using a well-established, mainly CD4^+^ T cell-driven animal model of neuroinflammation known as MOG_35–55_ peptide-induced EAE. Administration of a non-depleting NKG2D-blocking antibody has been shown to prevent the development of disease in murine models of autoimmunity (type 1 diabetes) [21,22], transfer-induced colitis [22], and collagen-induced arthritis [12]. This antibody blocks binding of NKG2D to its ligands, leading to internalization of the NKG2D receptor and thereby decreasing the proinflammatory potential also of CD4^+^NKG2D^+^ cells [22]. Compared with isotype-treated C57BL/6 mice, NKG2D-blocking antibody-treated mice showed a significantly ameliorated disease course of EAE (Figure 3A). Splenocytes isolated at disease onset from both groups exerted regular peripheral immune responses as shown by similar levels of proliferation and IFN-γ and IL-17 production upon re-stimulation with MOG (Figure 3B), suggesting that NKG2D blockade did not significantly interfere with the peripheral immune cell effector functions. Interestingly, the frequencies of brain infiltrating CD4^+^NKG2D^+^ or CD8^+^NKG2D^+^ T cells in NKG2D-blocking antibody-treated animals were reduced to basal conditions observed in naive mice (Figure 3C), pointing toward a reduction of cell migration into the CNS by NKG2D blockade. Consistent with the reduction of clinical symptoms, a significantly decreased number of inflammatory foci and smaller areas of demyelination were found in NKG2D-blocking antibody-treated mice, as assessed by immunohistochemical quantifications (Figure 3D). As an alternative approach to antibody treatment, a soluble NKG2D molecule (sNKG2D) was used in another set of EAE experiments. Administration of sNKG2D delayed disease onset and alleviated disease severity of EAE only when administered after immunization (Figure 3E, left panel), but not when sNKG2D was administered in the late phase of disease (Figure 3E, right panel). Taken together, inhibition of the NKG2D-signaling pathway has beneficial effects in an animal model of neuroinflammation.

### CD4^+^NKG2D^+^ T cells show cytolytic capacity towards cultured mouse oligodendrocytes

**Figure 4:**
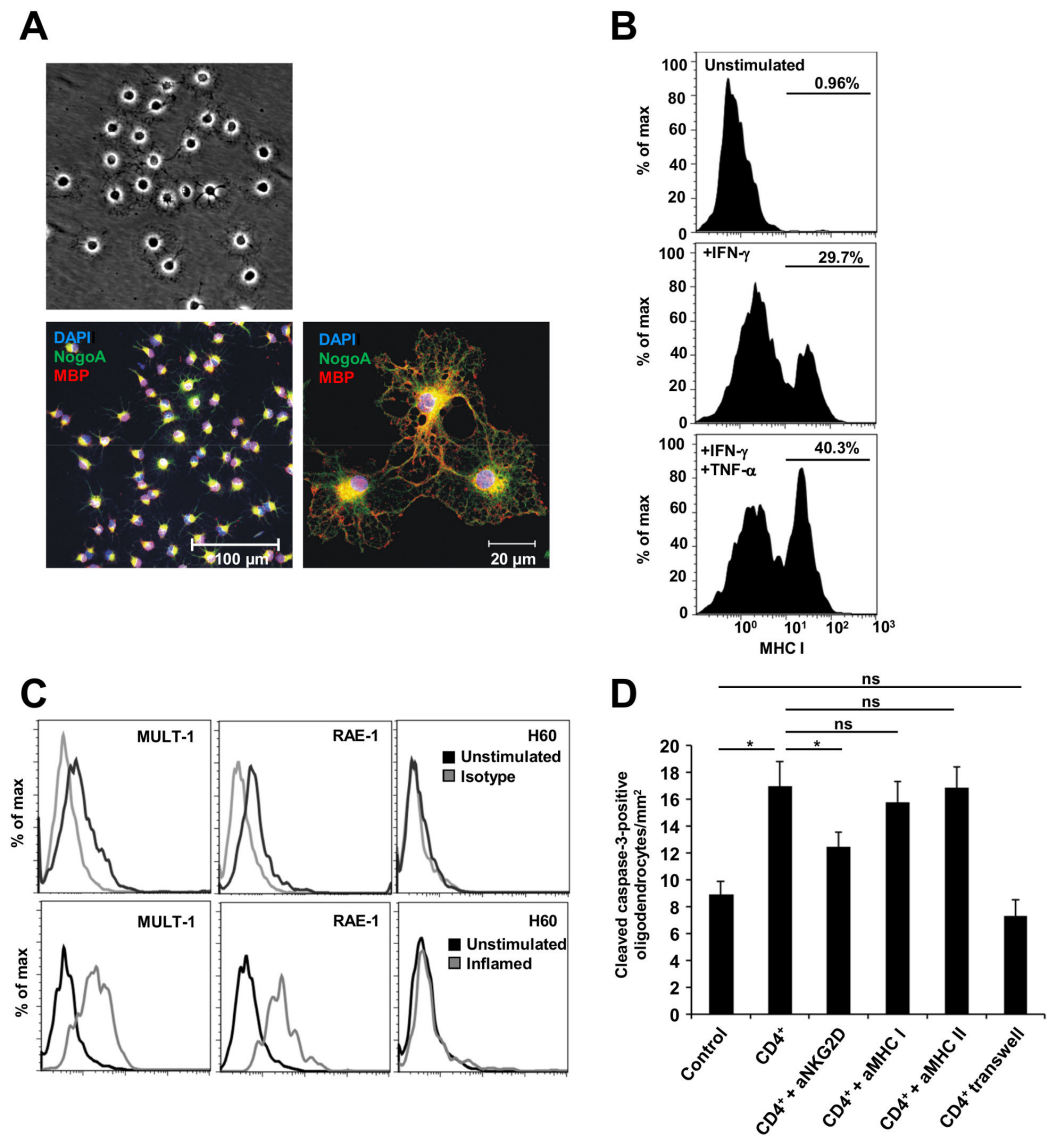
CD4^+^NKG2D^+^ T cells show cytolytic effects towards cultured mouse oligodendrocytes. (**A**) Upper panel: Oligodendrocytes after 48 h of differentiation (bright field). Lower panel: Immunocytochemistry of mature oligodendrocytes displaying a complex network of myelin-building processes (DAPI, blue; NogoA, green; myelin basic protein [MBP], red). (**B**) Flow cytometric quantification of MHC I expression of differentiated oligodendrocytes under basal and different inflammatory conditions (500 IU/ml IFN-γ for 24 h or 500 IU/ml IFN-γ and 500 IU/ml TNF-α for 24 h). (Representative example, *n* = 3). (**C**) Differentiated oligodendrocytes express the mouse NKG2D ligands MULT-1 and RAE-1, but not H60, under basal conditions. Upon inflammation (500 IU/ml IFN-γ and 500 IU/ml TNF-α for 24 h), the surface expression of these ligands is upregulated (*n* = 3). (**D**) The frequency of apoptotic oligodendrocytes is assessed by an immunohistochemical cleaved caspase-3 staining. Oligodendrocytes were co-cultured with CD4^+^NKG2D^+^-enriched CD4^+^ T cells (CD4^+^) for 6 h, and blocking antibodies against NKG2D, MHC I, and MHC II were added as indicated. In one assay, CD4^+^ T cells were separated by a Transwell inset (*n* = 3 independent experiments, triple values). *P < 0.05. ns, not significant.

To shed further light on the role of CD4^+^NKG2D^+^ cells in neuroinflammatory processes, we made use of mouse in vitro assays. Cytotoxic damage of oligodendrocytes is a key pathogenic feature of MS. Many different pathways are thought to be involved in this process. Among others, NKG2D signaling has been implicated in the cytotoxic responses mediated by NK cells, CD8^+^ T cells, and γδ T cells [19]. In order to elucidate a possible detrimental role of CD4^+^NKG2D^+^ T cells towards oligodendrocytes, we established mouse in vitro oligodendrocyte cultures. These cells formed a network of myelin-building processes and expressed markers of differentiation (Figure 4A). Under inflammatory stimuli (IFN-γ or IFN-γ and TNF-α), oligodendrocytes showed an upregulation of MHC I surface molecules as a marker of inflammatory responses (Figure 4B). Comparable with human endothelial cells, mouse oligodendrocytes expressed corresponding NKG2D ligands. MULT-1 and RAE-1, but not H60, were found under basal conditions and showed an upregulation upon inflammation (Figure 4C). To simulate the inflammatory conditions of MS lesions, we co-cultured pre-stimulated (IFN-γ and TNF-α) oligodendrocytes with CD3/CD28 pre-activated CD4^+^ T cells which were magnetic-activated cell sorting (MACS)-enriched for NKG2D (containing approx. 8–12% CD4^+^NKG2D^+^ T cells mimicking the human CSF data). The frequency of apoptotic oligodendrocytes was assessed by a cleaved caspase-3 staining (Figure 4D). The induction of apoptosis was dependent on cell-cell contact of T cells and oligodendrocytes as controlled in a transwell experiment. The blockade of NKG2D signaling, but not of MHC I or MHC II, led to a significant reduction of caspase-3-positive oligodendrocytes, supporting an antigen-independent but cell-contact dependent mechanism of action (Figure 4D). In summary, CD4^+^NKG2D^+^ cells were shown to be able to kill oligodendrocytes in an in vitro assay, which is in good agreement with their expression of cytolytic proteins (CD95, Granzyme B, perforin).

## Discussion

Different molecules and cell types provide a link between the innate and the adaptive immune system sharing characteristics of both systems. The best-known cell types are γδ T cells and NK T cells (characterized by an invariant TCR recognizing CD1d). NKG2D-expressing CD4^+^ lymphocytes (also named TNK cells by some authors [23]) differ profoundly from classical CD4^+^ T lymphocytes, as they possess NK-like properties, in addition to bearing a regular, polymorphic αβ TCR. Indeed, we show here that expression of the co-stimulatory receptor NKG2D defines a population of CD4^+^ T cells with elevated levels of markers for migration, activation, and cytolytic effector molecules. Under NKG2D co-stimulation CD4^+^NKG2D^+^ T cells demonstrate an increased cell proliferation compared to CD4^+^NKG2D^-^ T cells. Certain proportions of T cells were shown to proliferate in response to human myelin antigens in multiple sclerosis patients and healthy donors [24,25]. High concentrations of PLP_190-209_ lead to a preferential proliferative response of CD4^+^NKG2D^+^ cells. Furthermore, CD4^+^NKG2D^+^ cells produce high levels of IFN-γ and IL-17 upon stimulation.

Under non-pathologic conditions, NKG2D is already expressed on NK cells and a large part of CD8^+^ T cells. Only a small percentage of CD4^+^ T cells coexpresses NKG2D, but these cells correlate with disease in different autoimmune disorders. We therefore speculated that functional inhibition of NKG2D on CD4^+^ cells plays a major and significant role in attenuating the inflammatory response in MS. Indeed, we were able to demonstrate increased frequencies of CD4^+^NKG2D^+^ T cells in the CSF of RRMS patients compared with healthy controls with even higher frequencies in the active phases of MS, suggesting an association of NKG2D expression with disease activity. Increased frequencies of CD4^+^NKG2D^+^ T cells in the peripheral blood of RRMS patients with active disease might be based on either a peripheral proliferation or an elevated recirculation from the CNS. CD4^+^NKG2D^+^ T cells from MS patients expressed even higher levels of markers for migration, activation, and cytolytic effector molecules and produced higher levels of IFN-γ and IL-17 upon stimulation in comparison to CD4^+^NKG2D^+^ T cells from healthy donors. These findings point towards a MS specific increased potential of CD4^+^NKG2D^+^ T cells to migrate into the CNS and exert proinflammatory effector functions. Especially CCR 6 has been recently shown to be a brain-specific determinant for the constitutive trafficking of patrolling T cells into the CNS [26]. Furthermore CD4^+^NKG2D^+^ T cells in the CSF of MS patients showed a shift to an effector memory phenotype compared to healthy controls providing the ability of rapid maturation into effector T cells. Immunohistochemical staining of human brain tissue confirmed increased numbers of NKG2D-expressing CD4^+^ T cells in the perivascular regions of MS lesions, but not in healthy tissue samples or in normal-appearing white matter. This finding either reflects an enhanced migratory capacity of CD4^+^NKG2D^+^ T cells and/or an upregulation of NKG2D on CD4^+^NKG2D^−^ T cells in the inflammatory milieu of the CNS. We performed in vitro migration experiments that revealed an increased trafficking of CD4^+^NKG2D^+^ T cells with a clear dependency on T cell-endothelial cell interactions. Blockade or activation of NKG2D by antibodies modulated the migratory capacity of CD4^+^NKG2D^+^ T cells. Our findings point towards a yet unknown role of NKG2D for T lymphocyte migration.

Recent findings suggest a significant role of platelets in the pathogenesis of MS and EAE, e. g. the depletion of platelets led to an amelioration of EAE disease course [27]. Interestingly, platelet derived soluble factors have been shown to downregulate NKG2D on NK cells [28]. High concentrations of IL-15 in the inflammatory milieu of MS are able to counterbalance this effect [29,30] demonstrating the complex mechanisms involved in the regulation of the NKG2D signaling pathway. 

The destruction of oligodendrocytes by immune cells is a pathologic key finding in MS. Oligodendrocytes are known to be susceptible to MHC-independent cytotoxicity mediated by effector CD8^+^ T cells, γδ T cells, or NK cells [19]. This effect is at least partially dependent on NKG2D-NKG2D ligand interactions [19]. Accordingly, we observed a basal expression and an upregulation of NKG2D ligands on differentiated mouse oligodendrocytes in vitro. CD4^+^ T cells, enriched with CD4^+^NKG2D^+^ T cells to simulate the conditions of neuroinflammation, exerted an MHC I/II-unrestricted, but NKG2D- and cell-cell-contact-dependent, lysis of co-cultured oligodendrocytes.

To further enlighten the implications for the in vivo situation, we used a well-established, mainly CD4^+^ T cell-driven, mouse model of neuroinflammation known as MOG-EAE. Blockade of NKG2D by soluble NKG2D (sNKG2D) markedly delayed the onset of EAE when administered during the early phase of disease. In contrast, administering sNKG2D during the late phase of disease had no such effect, indicating that NKG2D blockade exerts beneficial effects predominantly in the phase of peripheral immune cell priming and immune cell trafficking to the CNS. Using an established NKG2D-blocking monoclonal antibody [21,22] in the early phase of EAE resulted in comparable findings. The histologic evaluation demonstrated significantly reduced demyelination and inflammatory foci. The frequency of CD4^+^NKG2D^+^ and CD8^+^NKG2D^+^ T cells was reduced to basal conditions after antibody treatment. In this respect, NKG2D inhibition had a considerably beneficial effect on neuroinflammation. It should be noted that the contribution of other NKG2D-expressing immune cell types cannot be formally ruled out in this animal model. Cell-type-specific NKG2D-knockout mice might overcome this issue in the future. Nevertheless, the NKG2D-dependent lysis of mouse oligodendrocytes by CD4^+^ T cells indicates a considerable involvement of CD4^+^NKG2D^+^ cells in demyelinating processes.

The limitations of our work are the small sample size of clinical samples owed to the rarity of suitable subjects, the limited transferability from observations made in mouse models to the human organism and the usage of a simplified model system to study the complex mechanisms at the blood-brain barrier. However, reductionist and simplified *in vitro* and *in vivo* models are the only instruments for an in-deep investigation of specific mechanisms.

In summary, our results identify CD4^+^NKG2D^+^ cells as a pro-inflammatory, pro-migratory and cytotoxic T helper cell subpopulation with potential impact on autoimmune CNS inflammation. Most importantly, our data show a yet undescribed and unexpected role of NKG2D in lymphocyte trafficking: NKG2D promotes transmigration over the BBB and CD4^+^NKG2D^+^ T cells show enhanced rates of migration upon NKG2D activation. Hence, the NKG2D-signaling pathway might be an important target for future drugs preventing inflammation and cell destruction. 

## Materials and Methods

### Ethics statement

All patients included in this study gave written informed consent in accordance with the Declaration of Helsinki and a protocol approved by the Ethics Committee of the University of Münster.

All animal experiments were approved by the local authorities (“Landesamt für Natur, Umwelt und Verbraucherschutz NRW; Fachbereich 87, Tiergesundheit, Tierschutz; Recklinghausen, Germany”) and conducted according to the German law of animal protection (87-51.04.2010.A263). All surgery was performed under deep isoflurane anesthesia, and all efforts were made to minimize suffering.

### Material from MS patients and healthy controls

Thirty-eight patients diagnosed with RRMS according to the McDonald criteria [31] were included in this study (23 with acute relapses, 15 with stable disease). Exacerbation was defined as the occurrence of a new symptom, or worsening of a pre-existing neurologic deficit, lasting at least 24 h after a period of stability or improvement of ≥ 30 days. MS patients who showed no new symptoms for at least 3 months prior to CSF and blood sampling (without gadolinium-enhancing lesions on magnetic resonance imaging) were considered clinically stable. Patients included in the study had not yet received immunomodulatory treatment except for corticosteroids, with the last dose at least 3 months prior to study. Patients with clinical relapses had no corticosteroid therapy at the time point of blood drawing or CSF examination. The control cohort was defined as age- and sex-matched patients without autoimmune or neuroinflammatory disorders (e.g. psychosomatic disorders or normal-pressure hydrocephalus; *n* = 15). 

### MS tissue brain specimens

For immunohistochemical stainings, human autopsy and biopsy material from MS patients and healthy controls was provided by The UK Multiple Sclerosis Tissue Bank (Division of Neuroscience and Mental Health, London, UK, *n* = 5) and the Department of Neuropathology, University Hospital Freiburg. For the present study, five MS cases were analyzed with available formaldehyde fixed, paraffin embedded brain tissue. The lesions fulfilled the morphologic criteria of an inflammatory demyelinating process consistent with MS when stained with hematoxylin and eosin (H&E), luxol fast blue-periodic acid-Schiff (LFB-PAS) myelin stain and Bielschowsky’s silver impregnation for axons. 

In brief, paraffin embedded tissue was deparaffinized and afterwards stained as follows. Slices were washed three times with PBS and incubated with 5% BSA (PAA, Cölbe, Germany), 1% normal goat serum (NGS; PAA) and 0.2% Triton X-100 (Sigma-Aldrich, Munich, Germany) for 1 h at room temperature. Afterwards, slices were incubated with primary antibodies diluted in PBS with 1% BSA and 1% NGS: Primary antibodies used for these stainings were polyclonal rabbit anti-human NKG2D (Bioss, Woburn, USA), monoclonal mouse anti-human CD4 and mouse anti-human CD8 (both Leica Biosystems, Wetzlar, Germany) antibodies overnight at 4°C. Slices were then stained with secondary antibodies (donkey anti-mouse Alexa Fluor^®^ 488 and goat anti-rabbit Alexa Fluor^®^ 555, both Life Technologies, Darmstadt, Germany) diluted in PBS with 1% BSA and 1% NGS for 1 h at room temperature. Primary antibodies were used at a 1:100 dilution and secondary antibodies were used at a 1:200 dilution. Staining with 0.5 μg/ml DAPI (Merck, Darmstadt, Germany) was performed for 5 min. Finally, slices were washed and subsequently covered with 1,4-diazabicyclo(2–2–2)octane (Merck, Darmstadt, Germany). 

For each patient, three slices within an MS lesion and eight areas of each slice were evaluated on a semi-quantitative scale by two blinded investigators (0, no CD4^+^NKG2D^+^ cells detectable; +, occasional NKG2D^+^CD4^+^ cells detectable, ++, NKG2D^+^CD4^+^ cells < 20%; +++, NKG2D^+^CD4^+^ cells > 20%). Negative controls were obtained by either omitting the primary or secondary antibody and revealed no detectable signal on subsequent analysis.

### Murine cell isolation and culture

Spleens were isolated from age- and sex-matched mice (aged 8–10 weeks). Tissue was homogenized and strained through a 40 µm nylon filter. The homogenates were rinsed with washing medium (DMEM containing 1% FCS, 1% glutamine, 1% antibiotics) and resuspended in erythrocyte lysis buffer (150 mM NH_4_Cl, 10 mM KHCO_3_, 0.1 mM EDTA; pH 7.3) for 2 min. For some experiments, immune cell subsets were isolated using appropriate magnetic bead-based separation kits (CD4^+^ T cell isolation Kit II, Miltenyi Biotec). Cells were cultured in DMEM containing 10 mM Hepes, 25 μg/ml gentamicin, 50 μM β-mercaptoethanol, 5% FCS, 2 mM glutamine, and 1% non-essential amino acids (Cambrex, Verviers, Belgium).

### Antibodies and reagents

The following primary anti-human antibodies were used for flow cytometry, the respective isotype controls were purchased from BD Biosciences (Heidelberg, Germany): CD4-FITC^1^, CD4-PerCP^1^, CD8-PerCP^1^, CD11a-APC^1^, CD44-FITC^1^, CD49d-APC^1^, CD56-APC^1^, CD62L-APC^1^, CD69-PerCP^1^, CD95-FITC^1^, CD107a-FITC^1^, CCR6-APC^1^, IFN-γ-FITC^1^, Granzyme-B-FITC^1^, MICA/B-PE^1^, TCRαβ-FITC^1^, CD8-PB^2^, CD31-FITC^2^, CD45RA-FITC^2^, HLA-DR-ECD^2^, CD54-PB^3^, Perforin-AF488^3^, Perforin-Alexa488^3^, CD28-FITC^4^, CD29-FITC^4^, IL-17A-PE^4^, NKG2D-APC^4^, CD3-PO^5^, CD4-APC^6^, CD25-APC^6^, NKG2D-PE^7^, ULBP-1 ^7^*, ULBP-2-APC^7^, and ULBP-3^7^*. (*Secondary labeled with goat anti-mouse-PE, Sigma-Aldrich, Munich, Germany ^1^BD Biosciences, Heidelberg, Germany ^2^Beckman Coulter, Krefeld, Germany ^3^Biolegend, Fell, Germany ^4^eBioscience, San Diego, CA, USA ^5^Invitrogen, Karlsruhe, Germany ^6^Miltenyi Biotec, Bergisch Gladbach, Germany; ^7^R&D Systems, Minneapolis, MN, USA). 

The following primary anti-mouse antibodies were used for flow cytometry, the respective isotype controls were purchased from BD Biosciences (Heidelberg, Germany): CD56-APC^1^, CD3-PE^2^, CD4-FITC^2^, H-2Kb-PE^2^, CD8a-AF700^3^, CD11b-APC^4^, H60-PE^5^, MULT1-PE^4^, NKG2D-FITC4^3^, RAE-1-PE^3^, and NKG2D-PE^4^. (^1^Abcam, Cambridge, UK ^2^BD Biosciences, Heidelberg, Germany ^3^Biolegend, Fell, Germany ^4^eBioscience, San Diego, CA, USA; ^5^R&D Systems, Minneapolis, MN, USA).

Activating anti-human NKG2D monoclonal antibody (clone 149810) was bought from R&D Systems (Minneapolis, MN, USA). Blocking humanized anti-NKG2D monoclonal antibody was obtained from Novo Nordisk (Måløv, Denmark). The corresponding isotypes were: mouse IgG1 (clone P3, eBioscience, San Diego, CA, USA) and human IgG4, κ (Sigma-Aldrich, Munich, Germany). IFN-γ and TNF-α were purchased from Peprotech EC Ltd. (London, UK). 

### Flow cytometry and cell sorting

Flow cytometric analysis of PBMCs (peripheral blood mononuclear cells) was performed as previously described [32]. Cells were analyzed on a BD FACS Calibur Flow Cytometer (BD Biosciences, Heidelberg, Germany) or a Gallios Flow Cytometer (Beckman Coulter, Krefeld, Germany). CD3^+^CD4^+^CD8^−^CD56^−^ cells were gated for NKG2D expression (further referred to as CD4^+^NKG2D^+^ or CD4^+^NKG2D^−^ within this article, for the gating strategy see Figure S1A).

For intracellular cytokine staining, the BD Cytofix/Cytoperm™ Fixation/Permeabilization Solution Kit with BD GolgiPlug™ was used (BD Biosciences, Heidelberg, Germany) according to standard protocols after stimulation with anti-human CD3 (OKT3; 2 µg/ml) and either soluble mouse anti-human CD28 (1 µg/ml; eBioscience, San Diego, CA, USA) or coated mouse anti-human NKG2D (5 µg/ml; clone MAB139; RD Systems) for 24 h. For some experiments, CD4^+^ T cells were stained with anti-human NKG2D-PE monoclonal antibody and sorted on a MoFlo™ XDP Cell Sorter from Beckman Coulter (Fullerton, CA, USA) or enriched using anti-PE microbeads (Miltenyi Biotec, Bergisch Gladbach, Germany).

### Transmigration assay

Transmigration assays in this in vitro model were performed as previously described [33,34]. Briefly, Transwell^®^ inserts (6.5 mm Transwell^®^ with a 3.0 µm pore polyester membrane insert; Corning Inc, Lowell, MA, USA) were coated with collagen IV (0.4 mg/ml; Sigma-Aldrich, Munich, Germany) and fibronectin (0.1 mg/ml) at 37°C for 1 h. The HBMECs (1.5 × 10^5^/insert) were added to the upper compartment. Cells were incubated (37°C, 5% CO_2_) overnight to allow them to attach to the membrane, thereby forming a single-layer. The quality of the HBMEC monolayer was monitored by measuring the transendothelial resistance and plasma membrane capacitance using a CellZscope system (nanoAnalytics, Münster, Germany) as described before [35]. Transmigration experiments were performed when high, stable resistance measurements indicated a confluent monolayer (for HBMEC typically 100-200 Ωxcm^2^, see also [35-37]). After reaching appropriate resistance values the supernatant was discarded and CD4^+^ T cells (5 × 10^5^/insert) were transferred to the HBMEC single-layer for a migration period of 6-12 h as indicated. Afterwards, a standardized amount of an unlabeled calibrite bead solution (BD Biosciences, Heidelberg, Germany) was added to the lower compartment of each well. Cell numbers were normalized by definition of a cell/bead ratio as previously described [35,38]. 

Migrated cells plus calibrite beads (lower compartment) and non-migrated cells (upper compartment) were harvested and washed twice with FACS (fluorescence activated cell sorting) buffer prior to flow cytometric analysis as described above.

### EAE experiments

EAE was induced by immunization of 8–10-week-old female C57BL/6 mice (Charles River, Sulzfeld, Germany) with 200 µg MOG_35–55_ peptide (Biotrend, Cologne, Germany). MOG peptide was added to complete Freund’s adjuvant to obtain a 1 mg/ml emulsion, which was injected subcutaneously at the flank of deeply anesthetized mice. Pertussis toxin was injected on the day of immunization and 2 days later at a dose of 400 ng (Alexis, San Diego, CA, USA). Mice were treated with an established NKG2D-blocking antibody (CX5; 150 µg/mouse i.p.; eBioscience, San Diego, CA, USA) or an isotype control (purified rat IgG1 κ, Biolegend, Fell, Germany) as indicated. In another set of experiments, animals were treated intraperitoneally with either sNKG2D (soluble NKG2D) or vehicle alone. For early-stage treatment, 100 µg sNKG2D was administered on the day of immunization and subsequently 50 µg on days 2, 4, 6, 8, and 10 post-immunization. For late-stage treatment, 100 µg sNKG2D was given on day 10 and subsequently 50 µg on days 12, 14, 16, 18, and 20. Groups were matched for age, weight, and sex, and mice were randomized to the control or treatment group. Scoring was done by a blinded observer using the following score system: 0, no abnormality; 1, limp tail tip; 2, limp tail; 3, moderate hind limb weakness; 4, complete paralysis of one hind limb; 5, mild paraparesis; 6, paraparesis; 7, paraplegia; 8, tetraparesis; 9, quadriplegia or premoribund state; and 10, death. 

### Statistical analysis

All results are presented as mean ± SEM. Statistical analysis was performed using a modified Student’s *t*-test in case of normally distributed data, or a Mann-Whitney test for parametric data without normality and equality of variance and for non-parametric datasets. A one-way ANOVA with Bonferroni post hoc test was used in the case of multiple comparisons for parametric data, and a Kruskal-Wallis ANOVA was used for non-parametric data P values < 0.05 were considered statistically significant.

See File S1 for further Materials and Methods.

## Supporting Information

Figure S1 **Flow cytometric gating strategy for CD4^+^NKG2D^+^ T cells.** (**A**) The dot blots depict the flow cytometric gating strategy for CD3^+^CD4^+^CD8^-^CD56^-^NKG2D^+^ (CD4^+^NKG2D^+^) T cells in the peripheral blood of a healthy donor. A staining with anti-NKG2D antibody and an isotype-control is shown. (**B**) The same gating strategy was used for CSF samples.
(TIF)

Figure S2 **NKG2D enhances the migratory capacity of CD4^+^NKG2D^+^ T cells in vitro.** (**A**) A model of the Transwell experimental set-up is depicted. The dot plot shows one representative experiment of the transmigration of MACS-purified CD4+ T cells through a non-inflamed single-layer of human brain microvascular endothelial cells (HBMECs; *n* = 5). (**B**) Fluorometric assessment of T-lymphocyte antigen-specific lysis assay of naive (*n* = 4) or inflamed (*n* = 3) HBMECs co-cultured with CD4^+^NKG2D^+^ or CD4^+^NKG2D^−^ T cells for 12 h. The effector to target ratio was 10:1; EC, endothelial cell.
(TIF)

Figure S3 **Characterization of NKG2D^+^ and NKG2D^-^ CD4^+^ T cells in healthy donors and MS patients.** (**A**) A representative example of the staining for CD4^+^NKG2D^+^ T cells in the peripheral blood and the cerebrospinal fluid (CSF) of a stable RRMS patient is depicted. (**B**) Flow cytometry staining of naive (CD45RA^+^CD62L^+^), T central memory (Tcm, CD45RA^-^CD62L^+^), T effector memory (Tem, CD45RA^-^CD62L^−^) and T effector memory RA (Tem-RA, CD45RA^+^CD62L^-^) CD4^+^NKG2D^+^ cells in the peripheral blood of RRMS patients (RRMS, n = 6) and healthy controls (HD, *n* = 6). (**C**–**F**) Mean fluorescence intensity (MFI) of different markers indicative for migratory capacity (**C**), activation (**D**), or cytolytic capacity (**E**, **F**) of CD4^+^NKG2D^−^ T cells from the peripheral blood of HDs (*n* = 6) or RRMS patients (*n* = 6). *P < 0.05. ns, not significant. (**G**) Representative CFSE proliferation assays of CD4^+^NKG2D^+^ T cells and CD4^+^NKG2D^−^ T cells under CD3/28 or CD3/NKG2D stimulation (*n* = 8).
(TIF)

Figure S4 **CD8+ T cells in the peripheral blood, in the CSF and in MS lesions expressed NKG2D in large part.** (**A**) Frequencies of CD8^+^NKG2D^+^ T cells in the peripheral blood and the cerebrospinal fluid (CSF) of patients with stable (*n* = 15) and active (*n* = 14) relapsing-remitting MS (RRMS) and healthy controls (*n* = 15) assessed by flow cytometry. (**B**) Histopathologic characterization of a representative human MS lesion (patient with RRMS) using antibodies directed against CD8 and NKG2D, a perivascular region is magnified showing CD8^+^NKG2D^+^ T cells (DAPI, blue; CD8, green; NKG2D, red).
(TIF)

File S1 **Supplementary Materials and Methods. Detailed information on further materials and methods applied in this study.**
(DOCX)
