# Correction: CD4^+^NKG2D^+^ T Cells Exhibit Enhanced Migratory and Encephalitogenic Properties in Neuroinflammation

**DOI:** 10.1371/annotation/e93fa4e6-ee8f-468a-8552-0e0aa505beaa

**Published:** 2013-12-20

**Authors:** Tobias Ruck, Stefan Bittner, Catharina C. Gross, Johanna Breuer, Stefanie Albrecht, Sabrina Korr, Kerstin Göbel, Susann Pankratz, Christian M. Henschel, Nicholas Schwab, Ori Staszewski, Marco Prinz, Tanja Kuhlmann, Sven G. Meuth, Heinz Wiendl

An organization funding the first author was incorrectly omitted from the Funding Statement. The Funding Statement should read: "This work was supported by the Transregio centre grant SFB 'Multiple Sclerosis' (B1 to N.S. and H.W.; B5 to S.G.M.), by the German Ministry for Research and Education (BMBF, UNDERSTANDMS, belonging to the 'German Competence Network of MS' to H.W. and M.P.), by the Interdisciplinary Center for Clinical Research (IZKF) Münster (SEED 03/12, to S.B., KuT3/006/11 to T.K. and Meu3/010/12 to S.G.M.), by the Genzyme Neuroimmunology Fellowship Program (to T.R.), and materials and a project grant by Novo Nordisk, Copenhagen, Denmark (to H.W.)." 

