# Supplementary figures and images for: CD4+NKG2D+ T Cells Exhibit Enhanced Migratory and Encephalitogenic Properties in Neuroinflammation
Source: PLoS One. 2013 Nov 25;8(11):e81455. doi: 10.1371/journal.pone.0081455 (PMC3839937; doi:10.1371/journal.pone.0081455)

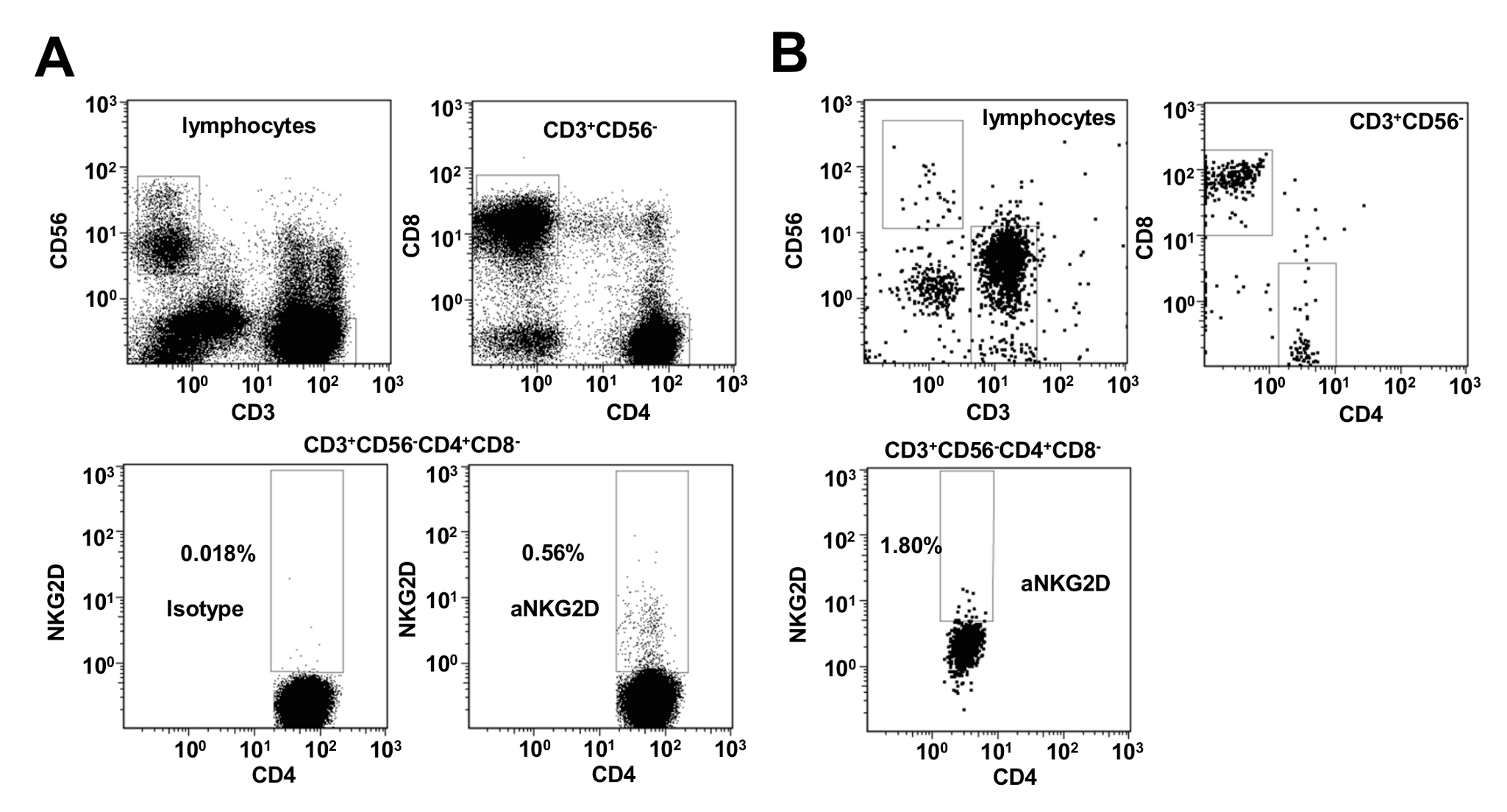

Supplement: Figure S1 — Flow cytometric gating strategy for CD4+NKG2D+ T cells. (A) The dot blots depict the flow cytometric gating strategy for CD3+CD4+CD8-CD56-NKG2D+ (CD4+NKG2D+) T cells in the peripheral blood of a healthy donor. A staining with anti-NKG2D antibody and an isotype-control is shown. (B) The same gating strategy was used for CSF samples. (TIF) [file pone.0081455.s001.tif]

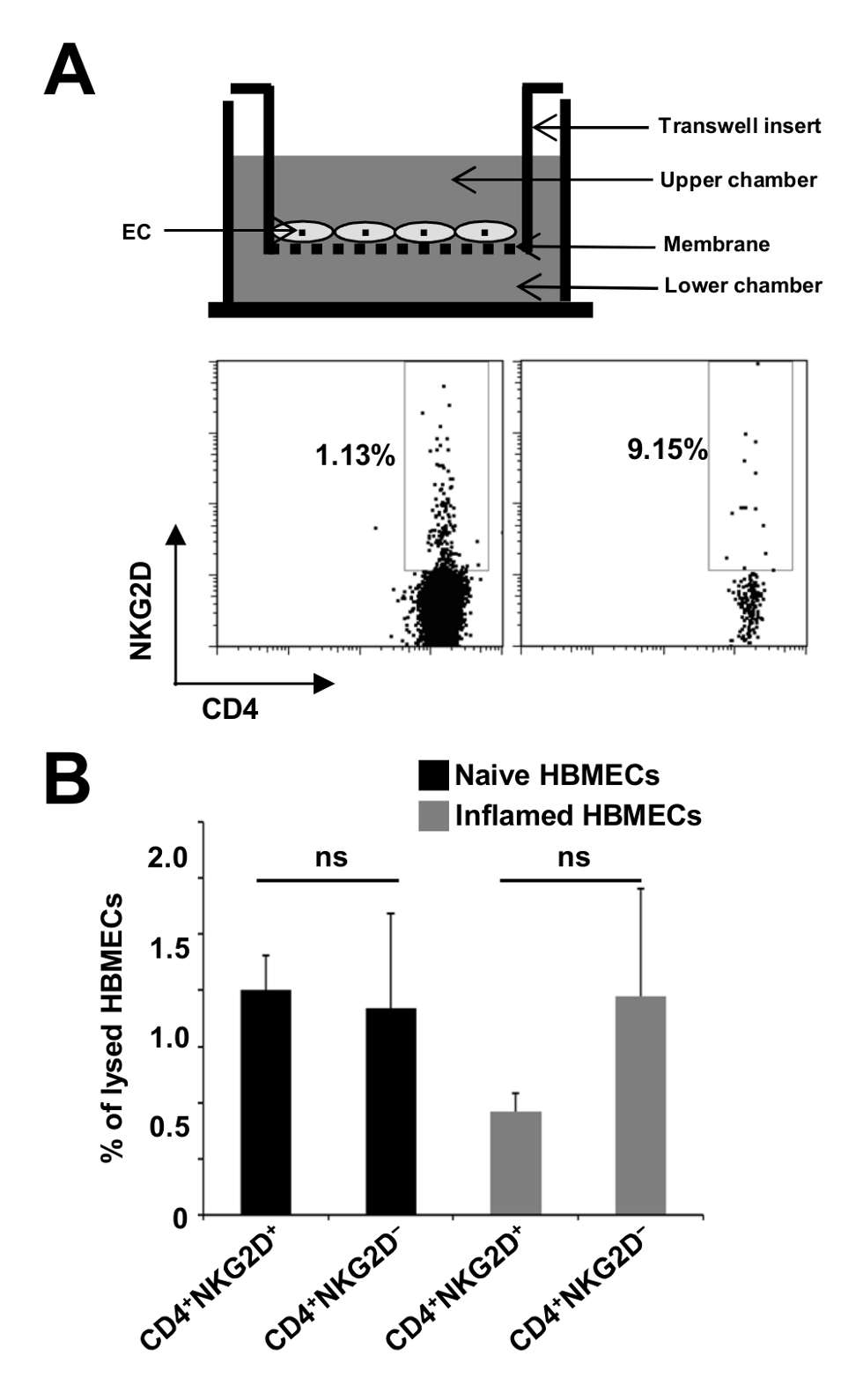

Supplement: Figure S2 — NKG2D enhances the migratory capacity of CD4+NKG2D+ T cells in vitro. (A) A model of the Transwell experimental set-up is depicted. The dot plot shows one representative experiment of the transmigration of MACS-purified CD4+ T cells through a non-inflamed single-layer of human brain microvascular endothelial cells (HBMECs; n = 5). (B) Fluorometric assessment of T-lymphocyte antigen-specific lysis assay of naive (n = 4) or inflamed (n = 3) HBMECs co-cultured with CD4+NKG2D+ or CD4+NKG2D− T cells for 12 h. The effector to target ratio was 10:1; EC, endothelial cell. (TIF) [file pone.0081455.s002.tif]

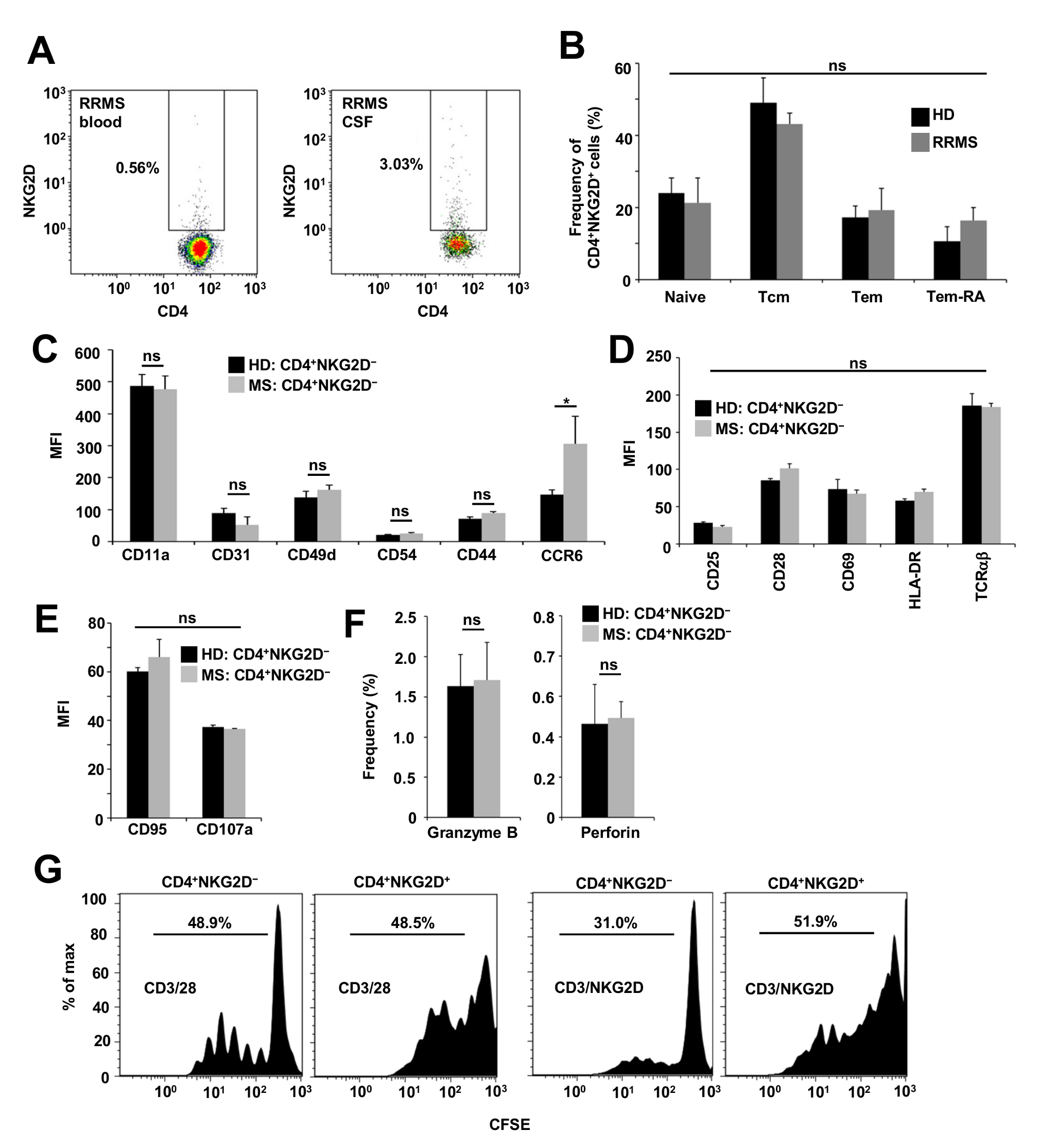

Supplement: Figure S3 — Characterization of NKG2D+ and NKG2D- CD4+ T cells in healthy donors and MS patients. (A) A representative example of the staining for CD4+NKG2D+ T cells in the peripheral blood and the cerebrospinal fluid (CSF) of a stable RRMS patient is depicted. (B) Flow cytometry staining of naive (CD45RA+CD62L+), T central memory (Tcm, CD45RA-CD62L+), T effector memory (Tem, CD45RA-CD62L−) and T effector memory RA (Tem-RA, CD45RA+CD62L-) CD4+NKG2D+ cells in the peripheral blood of RRMS patients (RRMS, n = 6) and healthy controls (HD, n = 6). (C–F) Mean fluorescence intensity (MFI) of different markers indicative for migratory capacity (C), activation (D), or cytolytic capacity (E, F) of CD4+NKG2D− T cells from the peripheral blood of HDs (n = 6) or RRMS patients (n = 6). *P < 0.05. ns, not significant. (G) Representative CFSE proliferation assays of CD4+NKG2D+ T cells and CD4+NKG2D− T cells under CD3/28 or CD3/NKG2D stimulation (n = 8). (TIF) [file pone.0081455.s003.tif]

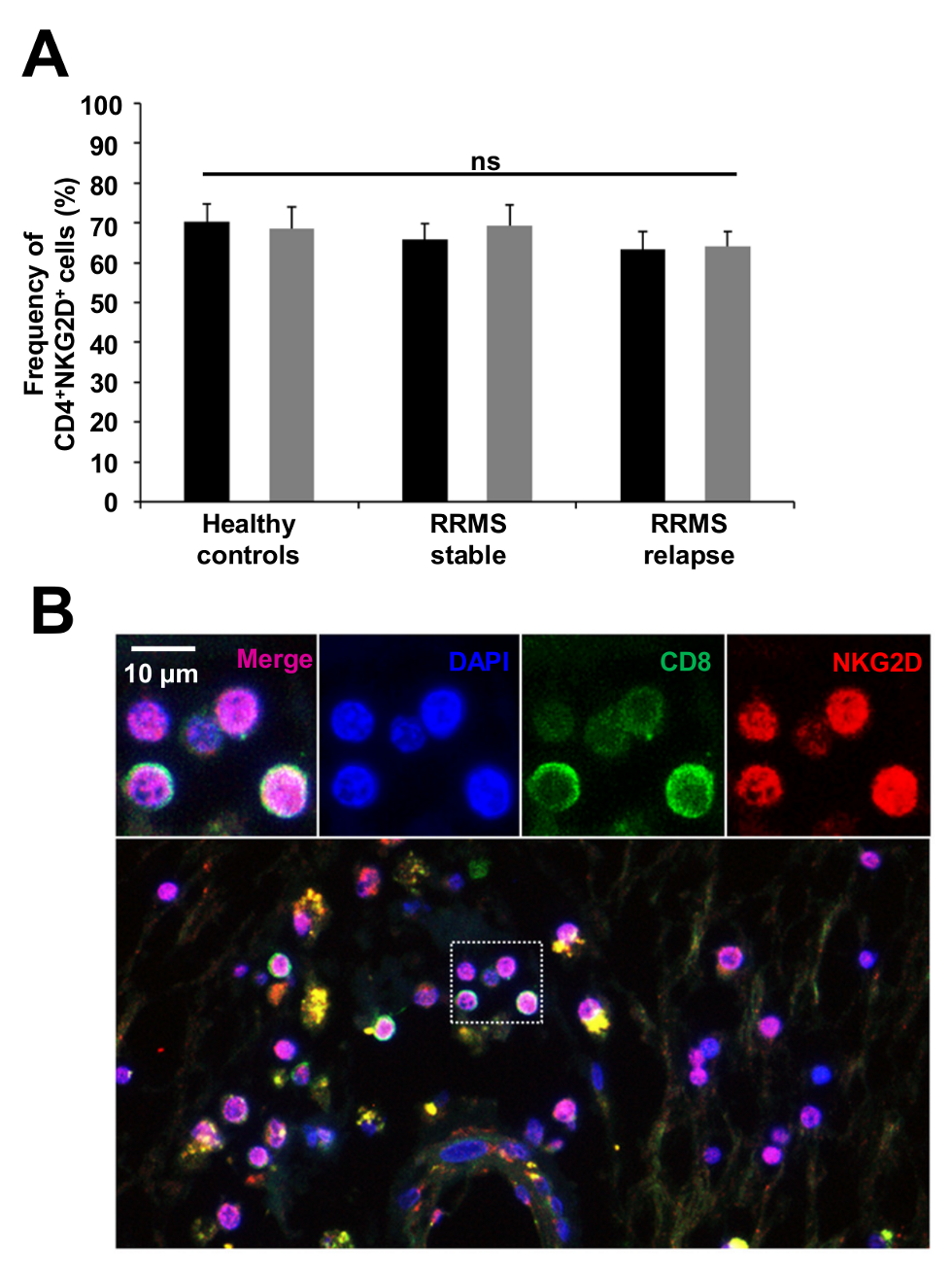

Supplement: Figure S4 — CD8+ T cells in the peripheral blood, in the CSF and in MS lesions expressed NKG2D in large part. (A) Frequencies of CD8+NKG2D+ T cells in the peripheral blood and the cerebrospinal fluid (CSF) of patients with stable (n = 15) and active (n = 14) relapsing-remitting MS (RRMS) and healthy controls (n = 15) assessed by flow cytometry. (B) Histopathologic characterization of a representative human MS lesion (patient with RRMS) using antibodies directed against CD8 and NKG2D, a perivascular region is magnified showing CD8+NKG2D+ T cells (DAPI, blue; CD8, green; NKG2D, red). (TIF) [file pone.0081455.s004.tif]
