# Supplementary material for: CD4+NKG2D+ T Cells Exhibit Enhanced Migratory and Encephalitogenic Properties in Neuroinflammation
Source: PLoS One. 2013 Nov 25;8(11):e81455. doi: 10.1371/journal.pone.0081455 (PMC3839937; doi:10.1371/journal.pone.0081455)
Supplement: File S1 — Supplementary Materials and Methods. Detailed information on further materials and methods applied in this study. (DOCX) [file pone.0081455.s005.docx]

**Supplementary Materials**

**Materials and Methods**

**Human cells and cell culture**

PBMCs were isolated from control patients and multiple sclerosis (MS) patients by centrifugation on a Lymphoprep™ (Fresenius Kabi Norge AS, Oslo, Norway) density gradient. For further experiments, CD4 T cells were negatively purified using the CD4 T cell Isolation Kit II (Miltenyi Biotec, Bergisch Gladbach, Germany) according to the manufacturer’s instructions. Cell purity was analyzed by flow cytometry and revealed > 98% of CD4 cells. Cell culture medium consisted of: RPMI 1640 (Gibco Invitrogen GmbH, Karlsruhe, Germany), FCS and penicillin/streptomycin (P/S) were from PAA (Cölbe, Germany). In one set of experiments, cells were labeled with 5 µM carboxyfluorescein succinimidyl ester (CFSE; Invitrogen, Karlsruhe, Germany) according to the manufacturer’s instructions, and proliferation was assessed by flow cytometry after stimulation with anti-human CD3 (OKT3; 2 µg/ml) and soluble mouse anti-human CD28 (1 µg/ml; eBioscience, San Diego, CA, USA), anti-human CD3 (2 µg/ml) and anti-human NKG2D (5 µg/ml, R&D Systems), human MOG_35-50_ (myelin oligodendrocyte glycoprotein, sequence: MEVGWYRPPFSRVVHLYRNGK, 10 µg/ml or 100µg/ml, AnaSpec, Fremont, CA, USA), human MBP_1-11_ (myelin basic protein, sequence: Ac-ASQKRPSQRHG, AnaSpec) and human PLP_190-209_ (proteolipid protein, sequence: SKTSASIGSLCADARMYGVL 10 µg/ml or 100µg/ml, AnaSpec) for 4 days. All experiments were performed in triplicate.

Cerebrospinal fluid (CSF) was obtained from patients by lumbar puncture and centrifuged within 30 min. The supernatant was discarded and cells were immediately analyzed by flow cytometry as previously described [[1](#_ENREF_1)]. Due to technical reasons, a cut-off of 8.000 lymphocytes was chosen; otherwise the sample was not used for evaluation (for the gating strategy see Fig. S1B).

Human brain microvascular endothelial cells (HBMECs; ScienCell Research Laboratories, Carlsbad, CA, USA) were cultured in fibronectin-coated (2 μg/cm^2^, Sigma-Aldrich, Munich, Germany) T-75 flasks with microvascular endothelial cell growth medium (Provitro, Berlin, Germany). Cells were harvested using Accutase™ (PAA, Cölbe, Germany) according to the manufacturer’s instructions, counted by CASY^®^ Model TT (Innovatis AG, Reutlingen, Germany) and used for experiments.

In one set of experiments, HBMECs or HBMECs stimulated for 24 h with 500 IU/ml IFN-γ and TNF-α were stained with CFSE (cytosolic dye) and DiD (membrane dye) and cocultured with CD4^+^NKG2D^+^ and CD4^+^NKG2D^−^ T cells for 12 h. CD4^+^NKG2D^+^ and CD4^+^NKG2D^−^ T cells were isolated from HLA-A2 mismatched alloreactive PBMCs by flow cytometric cell sorting.

**Oligodendrocyte cultures and killing assays**

Primary murine oligodendrocyte precursor cells (OPCs) were isolated according to the immunopanning method described earlier by Watkins et al. [[2](#_ENREF_2)], providing OPC single-cell cultures with a purity of 95%. In short, dissected forebrains from P6–P9 C57BL/6 mice were chopped in papain buffer, incubated for 20 min at 37°C and titrated in ovomucoid solution (CellSystems GmbH, Troisdorf Germany). For the initial negative selection, OPCs were centrifuged at 1,000 rpm for 10 min, resuspended in panning buffer and transferred to an anti-BSL 1 *Griffonia simplicifolia* lectin (L-110, Vector Labs, Burlingame, CA, USA) coated plate. Following a 15-min incubation at room temperature, the supernatant was transferred to a rat anti-mouse CD140a (Fitzgerald Industries International, North Acton, MA, USA) and AffiniPure goat anti-rat IgG (H1L) (Dianova, Hamburg, Germany) coated plate. After 45 min at room temperature, the supernatant was aspirated and bound OPCs were detached by subsequently using Trypsin and Earle's balanced salt solution, pH 6. Cells were centrifuged as described above, resuspended in OPC Sato media and plated in poly-L-lysine-coated T75 flasks. Isolated OPC were propagated at 37°C/5% CO_2_, with daily supplementation of platelet-derived growth factor AA (PDGF-AA; 10 µg/ml) and half the medium changed every 2–3 days. To induce differentiation, PDGF-AA was replaced by ciliary neurotrophic factor (10 µg/ml). Purity of differentiated oligodendrocytes was assessed by immunocytochemistry. After 48 h of differentiation, cultured oligodendrocytes were fixed with 4% PFA/PBS for 20 min. Oligodendrocytes were washed twice with PBS, and saturated for 15 min with 5% FCS/PBS. As primary antibodies, rabbit anti-MBP (1:100; DAKO, Hamburg, Germany) and mouse anti-NogoA (1:10.000; clone 11c7) were used and incubated overnight at 4°C. After three washing steps with PBS, the secondary antibodies goat anti-rabbit Cy3 and goat anti-mouse Cy2 were applied for 2 h at room temperature. After three washing steps with PBS, the cells were mounted with DAPI containing mounting medium to visualize the cell nuclei.

CD4^+^ were isolated 2 days before and stimulated with coated hamster anti-mouse CD3ε (clone 145-2C11, 2 µg/ml, BD Biosciences, Heidelberg, Germany), soluble hamster anti-mouse CD28 (clone 37.51, 1 µg/ml, BD Biosciences, Heidelberg, Germany), and IL-2 (100 IU/ml), and enriched with NKG2D-PE and anti-PE microbeads as described above. Antibodies were titrated yielding an enrichment of about 10% CD4^+^NKG2D^+^ cells. Cells were used in an effector to target cell ratio of 10:1 for 6 h. In a subset of experiments, an NKG2D-blocking antibody (CX5; 10 µg/ml, eBioscience), a respective isotype control (rat IgG1 κ, 10 µg/ml, Biolegend, Fell, Germany), an MHC I-blocking antibody (SF1-1.1.1, 20 µg/ml, eBioscience), an MHC II-blocking antibody (M5/114.15.2, 10 µg/ml, eBioscience), or Transwell^®^ inserts (Corning Inc., Lowell, MA, USA) were used. Histologic staining was performed as described below using cleaved caspase­3 rabbit anti-mouse and Cy3 goat anti-rabbit antibodies. Evaluation was performed by a blinded investigator.

**Immunohistochemical analysis of EAE experiments**

For histologic evaluation of EAE, mice were transcardially perfused with PBS. Spinal cords were carefully excised and embedded in Tissue-Tek® OCT compound (Miles Laboratories, Elkhart, USA). Ensuring that the same lumbar region was analyzed for all mice, 10-µm transverse cryosections were cut and stained with H&E or LFB according to standard protocols. Ten tissue sections, each section being at least 40 µm apart, were analyzed from five animals per group. Histologic quantifications were visually counted by a blinded investigator using a microscope (Axiophot2, Zeiss, Oberkochen, Germany) equipped with a charge-coupled device camera and analyzed MetaVue® Software (Molecular Devices, Downington, PA, USA).

**Immunologic analysis of EAE experiments**

Splenocytes from myelin oligodendrocyte protein (MOG)-immunized mice were restimulated with MOG peptide (10 µg/ml, same stock as used for immunization) and IFN-γ levels were assessed by ELISA (R&D Systems, Wiesbaden, Germany). For the evaluation of cell proliferation, the amount of ATP in the supernatant following cell lysis was assessed as a parameter of cell proliferation using the ATPLite™ Luminescence Assay System (PerkinElmer) according to the manufacturer’s instructions. Luminescence was measured on a TopCount NXT™ (PerkinElmer) and the splenocyte stimulation index (cell abundance with stimulation divided by cell abundance without stimulation) was calculated. For flow cytometric evaluation of central nervous system-invading cells, mice were perfused transcardially with PBS to diminish contamination by leukocytes located within blood vessels. Central nervous system tissue was dissociated mechanically and mononuclear cells from the interface of a 30–50% Percoll (Amersham, Piscataway, NJ, USA) density centrifugation were counted by Casy^®^ Model TT and stained with appropriate antibodies.

**Production of soluble mouse NKG2D (sNKG2D)**

Recombinant soluble mouse NKG2D lacking both the amino-terminal cytoplasmic region and the transmembrane domain was produced in Sf9 insect cells (Invitrogen, Karlsruhe, Germany) using the BAC-to-BAC^®^ system (Gibco Life Technologies, Paisley, UK). The expression construct included an amino-terminal FLAG^®^ (Sigma-Aldrich, Munich, Germany)/hexahistidine [[3](#_ENREF_3),[4](#_ENREF_4)].

1. Huang YH, Zozulya AL, Weidenfeller C, Metz I, Buck D, et al. (2009) Specific central nervous system recruitment of HLA-G(+) regulatory T cells in multiple sclerosis. Ann Neurol 66: 171-183.

2. Watkins TA, Emery B, Mulinyawe S, Barres BA (2008) Distinct stages of myelination regulated by gamma-secretase and astrocytes in a rapidly myelinating CNS coculture system. Neuron 60: 555-569.

3. Friese MA, Platten M, Lutz SZ, Naumann U, Aulwurm S, et al. (2003) MICA/NKG2D-mediated immunogene therapy of experimental gliomas. Cancer Res 63: 8996-9006.

4. Steinle A, Li P, Morris DL, Groh V, Lanier LL, et al. (2001) Interactions of human NKG2D with its ligands MICA, MICB, and homologs of the mouse RAE-1 protein family. Immunogenetics 53: 279-287.
